# Role of Sphingolipid Signaling in Glomerular Diseases: Focus on DKD and FSGS

**DOI:** 10.33696/Signaling.1.013

**Published:** 2020-09

**Authors:** Alla Mitrofanova, Yelena Drexler, Sandra Merscher, Alessia Fornoni

**Affiliations:** 1 Katz Family Division of Nephrology and Hypertension, Department of Medicine, University of Miami, Miller School of Medicine, Miami, Florida, USA; 2 Peggy and Harold Katz Family Drug Discovery Center, University of Miami, Miller School of Medicine, Miami, Florida, USA; 3 Department of Surgery, University of Miami, Miller School of Medicine, Miami, Florida, USA

**Keywords:** Kidney, DKD, FSGS, Podocyte, Sphingolipids, SMPDL3b, S1P, C1P, Ceramide

## Abstract

Sphingolipids are well-recognized as major players in the pathogenesis of many human diseases, including chronic kidney disease. The kidney is a very sensitive organ to alterations in sphingolipid metabolism. The critical issues to be addressed in this review relate to the role of sphingolipids and enzymes involved in sphingolipid metabolism in the pathogenesis of glomerular diseases with a special focus on podocytes, a key cellular component of the glomerular filtration barrier. Among several sphingolipids, we will highlight the role of ceramide, sphingosine, sphingosine-1-phosphate and ceramide-1-phosphate. Additionally, we will summarize the current knowledge with regard to the use of sphingolipids as therapeutic agents for the treatment of podocyte injury in kidney disease.

## Introduction

**Figure 1:**
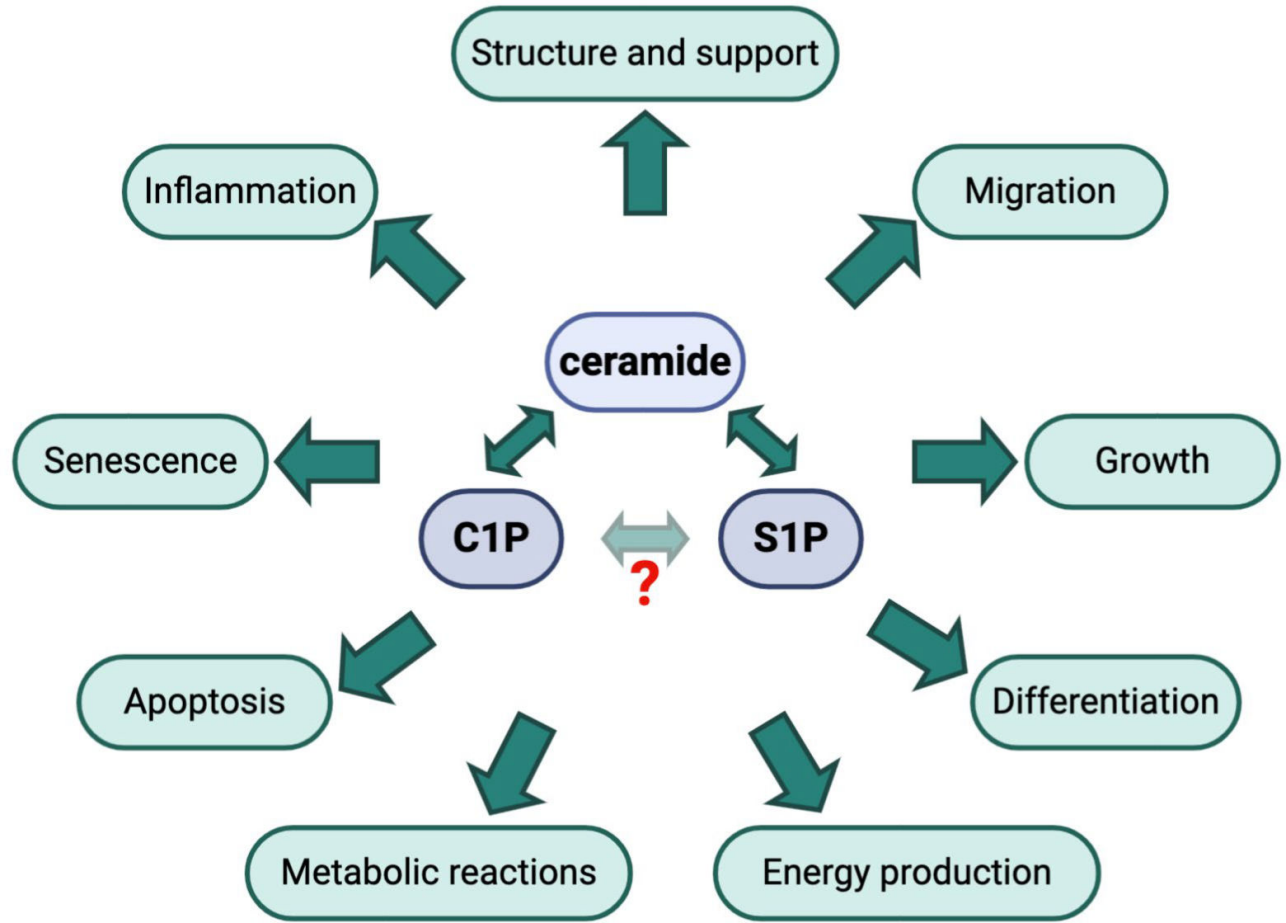
The biological roles of active sphingolipid metabolites. Ceramide, sphingosine-1-phosphate (S1P) and ceramide-1-phosphate (C1P) can be converted into each other and regulate many important functions in a cell. While the conversion of C1P to S1P and vice versa might be possible, it has not yet been confirmed experimentally.

Being a sophisticated and highly organized living system, mammals harbor a large number of biomolecular machineries which represent a dynamic and complex network of interconnections responsible for the effective operation, development and survivability of their body cells. Sphingolipids are a special class of lipids in eukaryotic cells, which have recently gained the attention of researchers because of their involvement in several fundamental processes of living cells, including proliferation and cell death (Figure 1).

Within the kidney, the proper filtration function relies primarily on podocytes, which are terminally differentiated epithelial cells lining the urinary surface of the glomerular capillary tuft. Changes in the function and number of podocytes can lead to the development and progression of glomerular diseases, including diabetic kidney disease (DKD) and focal segmental glomerulosclerosis (FSGS), both of which are common causes of end-stage kidney disease (ESKD) in the USA [1]. However, the cause of podocyte injury and detachment in DKD and FSGS remains largely unknown. Proper filtration function of the glomerular filtration barrier depends heavily on the integrity of lipid raft domains, special regions of the plasma membrane which are enriched in cholesterol, sphingomyelin and glycosyl-phosphatidylinositol (GPI)anchored proteins. However, renal accumulation of lipid droplets observed both in clinical and experimental glomerular disorders has been shown to correlate with the development of glomerulosclerosis [2–4]. Additionally, seminal studies in experimental DKD established a clear role of glycosphingolipids in its pathogenesis [5]. More recently, podocyte malfunction due to altered metabolism of ceramide-1-phosphate [6, 7] and sphingosine-1-phosphate [8–11] has been described to directly contribute to podocyte injury. In this review we will discuss the contribution of sphingolipids to the pathogenesis of glomerular diseases with a major focus on DKD and FSGS.

## Glomerular Diseases: Focus on DKD and FSGS

Chronic kidney disease (CKD) is a condition of gradual loss of kidney function. In 2019, it was estimated that 37 million people, or 15% of US adults, are affected by CKD [12]. Major CKD risk factors include diabetes and high blood pressure, while obesity, heart disease, family history of CKD, and older age may also contribute significantly to kidney damage. Current treatment strategies may slow the decline in kidney function and delay kidney failure, but do not prevent CKD progression. Thus, the development of new treatment options is critical to cure CKD.

### Diabetic kidney disease (DKD)

DKD is a major cause of chronic kidney disease leading to ESKD [1]. Clinically, one of the early features of DKD is loss of podocytes. Podocyte loss is an independent predictor of DKD progression in patients with type 1 (T1D) and type 2 (T2D) diabetes [13,14], resulting in a compromised filtration barrier and leakage of proteins into the urine (proteinuria). Several stimuli, such as hyperglycemia, chronic inflammation, oxidative stress and hemodynamic changes have been shown to contribute to podocyte injury in DKD. More recently, altered renal metabolism of lipids such as cholesterol, triglycerides [2,15–17], and sphingolipids has been shown to negatively impact podocyte function and lead to DKD progression and will be reviewed here.

### Focal and segmental glomerulosclerosis (FSGS)

FSGS is a histologic lesion that manifests clinically with nephrotic syndrome and is characterized by the presence of sclerotic lesions that affect some glomeruli (focal) and only some area of each glomerulus (segmental). FSGS is the most common cause of primary glomerular disease in adults in the United States and accounts for 10–15% of nephrotic syndrome cases [18]. Irrespective of the form of FSGS, whether primary (idiopathic), secondary, or genetic, loss of podocytes is an important determinant of progression to ESKD [19]. Among different causes of FSGS, recently described familial forms of FSGS demonstrate a causative link between altered sphingolipid metabolism and FSGS [8,9].

## Sphingolipid Signaling in Glomerular Disease

### Overview of sphingolipid metabolism: focus on the most important metabolites

Sphingolipids were long thought to be passive barrier lipids in cell membranes. Only in the last two decades sphingolipids and their metabolites, the most important being ceramide, sphingosine, ceramide-1-phosphate, and sphingosine-1-phosphate, have been considered as bioactive signaling molecules.

Sphingolipids belong to a large and diverse class of lipids that share a sphingosine base backbone, which is linked to a fatty acid (usually palmitic and stearic acid) via an amide bond, and are known to regulate cell membrane fluidity and structure [20]. The most critical role sphingolipids play in lipid rafts or raft-related caveolae, which are sphingomyelin- and cholesterol-rich microdomains of the plasma membrane, is to regulate protein-protein interactions and intracellular signal transduction. Thus, ceramide accumulation at the plasma membrane results in changes to the biophysical properties of the cell membrane, leading to altered lipid raft composition and changes in signaling properties (as reviewed in Ref. [21]).

Ceramide (Cer) is the central lipid intermediate of sphingolipid metabolism and regulates many of the functions in a cell, particularly anti-proliferative responses, such as growth arrest and/or senescence, and apoptosis (Figure 1). Additionally, ceramides are recognized as tumor-suppressive lipids and the inhibition of ceramidases has proven effective in anticancer therapy [22]. Ceramides can be produced by *de novo* synthesis from sphingosine (on the surface of the endoplasmic reticulum through the condensation of L-serine and palmitoyl-CoA by serine palmitoyl transferase) by the salvage pathway or from the hydrolysis of sphingomyelin (SM) or other complex sphingolipids by sphingomyelinases [23].

Ceramide kinase (CERK) catalyzes the formation of another bioactive lipid, ceramide-1-phosphate (C1P), from ceramide. The first biological role of C1P was reported in 1995 by Gomez-Munoz et al., who established a role for C1P as a regulator of cellular proliferation and growth [24]. Later on, C1P was described as an anti-apoptotic lipid [25] and, currently, it is well recognized as an important regulator of inflammation (reviewed in Ref. [26]). C1P is also involved in the production of pro-inflammatory eicosanoids, recruiting enzymes responsible for the release of the eicosanoid precursor arachidonate to the plasma membrane [27]. Interestingly, a hypothetical possibility for the direct conversion of C1P to S1P and vice versa has also been suggested [28].

Sphingosine (Sph) is generated from ceramide by the action of ceramidases, primarily alkaline ceramidase 1 (ACER1), and is subsequently converted to sphingosine-1-phosphate (S1P) by the action of sphingosine kinases (type 1 and type 2). S1P is implicated in cellular growth, survival, migration, angiogenesis and immune reactions (Figure 1). S1P can be dephosphorylated to sphingosine by S1P phosphatase or irreversibly degraded by S1P lyase leading to the formation of ethanolamine-1-phosphate and C16 fatty aldehyde. Interestingly, in the kidney, S1P lyase has been shown to play a role in development of proteinuria in mice [10], while genetic mutations in the gene coding for S1P lyase are associated with severe podocyte injury and nephrotic syndrome in humans [8,9]. Another interesting and important peculiarity of S1P is that it operates both intra-and extra-cellularly, i.e. as an autocrine and paracrine agent [29]. As an autocrine agent, S1P acts through five specific G protein-coupled receptors (designated S1P_1_-S1P_5_) and triggers distinctive signaling pathways and cellular responses, some of which can be antagonistic. S1P is primarily produced by erythrocytes and its concentration in blood and tissue may represent a biomarker for some diseases [30–34].

Over the last decades, little attention has been paid to the role of sphingolipids in the kidney and, to their role in podocyte function in health and disease. However, more recently, researchers have actively investigated the role of sphingolipids in the kidney, particularly in glomerular diseases. The accumulation of sphingolipids in glomerular diseases may be of genetic or non-genetic origin. For more information on the role of sphingolipids in glomerular diseases of genetic origin, readers are referred to a review previously published by us [35].

### Sphingolipids in DKD

Historically, increased levels of glycosphingolipids [36], ceramide [37], sphingosine and sphinganine [38] have been reported in the plasma of patients with T1D and T2D.

A recent study performed on diabetic C57BLKS *db/db* mice demonstrated elevated long-chain ceramides (C14:0, C16:0, C18:0, C20:0) and C18:0 glucosylceramide in plasma, while long-chain (C14:0, C16:0, C18:0) and very-long-chain (C24:0, C24:1) ceramide species and C16:0 glucosylceramide levels were decreased in kidney cortices [39]. In the same study, microarray analysis of kidney cortex from 24-week-old diabetic mice showed significantly altered expression of genes involved in ceramide synthesis and metabolism (decreased expression of *Degs2*, *Cers5*, *Fads3*, *Smpd2*, and increased expression of *Sptlc2*, *Cers4*, *S1Pr1*, *Acer2*, *Smpdl3b*). Similarly, our studies also demonstrated decreased levels of total ceramide in kidney cortices of *db/db* mice [6,40], where long-chain species (C14:0, C18:0, C18:1, C20:0, C20:1), but not very-long-chain species were affected [6]. In a prospective study on T2D patients from Singapore, long-chain ceramide levels (C16:0 and C18:0) were also found elevated in the plasma of patients with early or overt DKD compared to non-DKD controls [41]. Interestingly, high plasma levels of very-long-chain ceramide species were associated with a decreased risk of progression to macroalbuminuria in a subgroup of T1D patients enrolled in the Diabetes Control and Complications Trial (DCCT) and its long-term observational follow-up (EDIC) [42], which may reflect a regulatory role of ceramides in loss of renal function. On the other hand, podocyte-specific deletion of the main catalytic subunit of acid ceramidase resulted in ceramide accumulation in glomeruli and development of nephrotic syndrome in mice [43]. Notably, a recent report on urinary sphingolipids from patients with DKD also showed elevated urinary levels of ceramide d18:1/16:0, d18:1/18:0, d18:0/20:0, d18:1/22:0 and d18:1/24:0, which were correlated with urinary albumin [44]. It should be noted that increased levels of ceramide species were observed in diabetic patients with CKD stage 4, which may be due to the progression of kidney injury and reduced synthesis and/or excretion of ceramides by the kidney. Indeed, production of very-long-chain ceramide species is regulated by ceramide synthases (CerS), where CerS2, the most highly expressed isoform in the kidney, has been found to be associated with albuminuria in diabetic patients [45], and its haploinsufficiency has been reported to induce insulin resistance in the liver of mice fed on a high fat diet (HFD) [46].

In addition, ceramides have gained recent attention due to their role in insulin resistance, a significant contributor to DKD development and progression (as reviewed by us previously in Ref. [47]). Ceramides are known to inhibit signal transduction via phosphatidylinositol-3 kinase and block activation of protein kinase B (AKT), thereby interfering with glucose uptake and impairing storage of glycogen and triglycerides (as reviewed in Ref. [48]). In a recent study using diabetic mice, deletion of dihydroceramide desaturase 1 (DEGS1), an enzyme critical to the formation of ceramide from dihydroceramide, resolved hepatic steatosis and insulin resistance [49]. Similarly, a beneficial effect of pharmacological or genetic ablation of DEGS1 was shown to be associated with decreased obesity-associated lipid accumulation in adipocytes [50]. In a deterministic and stochastic simulator model using cultured macrophages, ceramide and S1P were shown to play a role in controlling the AKT pathway and insulin resistance via manipulating levels of ceramide synthases (CERS2, CERS5 and CERS6) and DEGS2 [51]. In MIN6 cultured cells, elevated levels of ceramides (C14:0, C16:0, C20:1, C24:0) were associated with decreased insulin secretion and increased apoptosis [52], indicating the undeniable involvement of ceramides in development of insulin resistance. The role of ceramides in insulin resistance is reviewed elsewhere in greater detail [53]. Furthermore, we have recently identified a sphingolipid related enzyme that affects insulin receptor (IR) subtype signaling (as reviewed more extensively below).

Ceramide can be further catabolized to sphingosine, which is then phosphorylated to S1P, another important bioactive sphingolipid. Recent studies support an important role for S1P in renal diseases, including renal fibrosis (reviewed in Ref. [54]), nephrotic syndrome [8,9] and other glomerular diseases such as IgA nephropathy and lupus nephritis [55,56]. Early studies of streptozotocin (STZ)-treated rats revealed increased levels of S1P in isolated glomeruli during initial stages of DKD [57]. In support of this observation, another study also demonstrated increased renal levels of S1P in mice with STZ-induced DKD, which was prevented by intraperitoneal injections of insulin [58].

As mentioned earlier, S1P may act as an extra- or intracellular signaling molecule. Extracellular S1P is synthesized in the cytosol by SphK1/Sphk2 and then exported by a number of transporters. Such transporters include SPNS2, MFSD2B, and the ATP-binding cassette (ABC) transporters ABCA, ABCC1, and ABCG2. We previously reported that ABCA1 deficiency is one of the peculiarities in glomeruli isolated from patients with T2D and DKD [2], an observation which we further confirmed by demonstrating that genetic or pharmacological overexpression of ABCA1 is sufficient to ameliorate podocyte injury in a mouse model of DKD [16,17]. Unbalanced expression of S1P receptors (enhanced S1PR2 expression and decreased S1PR1 expression) in mesangial cells of STZ-induced diabetic rats has been also shown to contribute to DKD progression [59]. Increased expression of S1P has been shown to be associated with increased reactive oxygen species production and injury in CKD [60].

On the contrary, much less is known about the role of C1P in DKD development and progression. C1P has been found to modulate AKT phosphorylation in skin fibroblasts, hematopoietic cells [61], macrophages [62,63], and adipocytes [64], consistent with the observation that bioactive sphingolipids are major modulators of insulin signaling [65–67]. Knockdown of ceramide kinase was demonstrated to be efficient in the treatment of mesangioproliferative glomerular diseases [68]. Our studies demonstrated that diabetic *db/db* mice have less total C1P (with C18:0 species mostly affected) in kidney cortices, and that exogenous administration of C1P protects from DKD progression [6].

It is important to note that other sphingolipids, such as glucosylcerebrosides (GlcCer) and gangliosides (especially GM3), have been previously reported to contribute to DKD development [69–71]. GM3 is the most abundant renal ganglioside. In podocytes, GM3 has been shown to localize in lipid rafts, which are also called GM3-raft domains, and to bind the soluble vascular endothelial growth factor receptor sFLT-1, which plays a critical role in the regulation of angiogenesis and rapid actin reorganization [72]. Moreover, a pivotal role of GM3 in promotion of insulin resistance has been demonstrated [73]. In patients with DKD, increased levels of sialic acid, one of the components of gangliosides, were significantly positively correlated with hemoglobin A1c, serum creatinine, and microalbuminuria [74]. The biology and role of GM3 in diabetes and other metabolic-related diseases is reviewed elsewhere [75]. Levels of glucosylceramide (GlcCer) in diabetic *ob/ob* mice and STZ-induced diabetic rats are increased in several tissues, including liver, muscle [76] and kidney [69]. Pharmacological inhibition of glycosphingolipid synthesis has been shown to have a beneficial effect on insulin sensitivity and glycemic and weight control in animal models of obesity and diabetes [76–78]. In diabetic *db/db* mice, elevated levels of hexocyl-, glucosyl-, galactosyl- and lactosylceramides were shown in kidney cortices [79]. In addition, a recent study reported that C18:1 hexosylceramide is associated with DKD, while very-long-chain lactosylceramides are associated with the development of microalbuminuria in patients with T1D [80].

### Sphingolipids in FSGS

Compared to DKD, much less is known about the role of sphingolipids in FSGS development and progression. In a mouse model of FSGS (doxorubicin-induced nephropathy), lipid peroxidation was reported as the main driver of macrophage-derived foam cell formation [81]. Using a mass spectrometric metabolomics approach, a recent report demonstrated that the urine of patients with FSGS contains elevated levels of fatty acids (C16:0, C22:4) and lysophosphotidylcholines (C14:0, C18:1) and decreased levels of phosphotidylcholine (C38:4) when compared to healthy subjects [82]. The same study also reported decreased levels of urinary acylcarnitine (C12:0) in patients with FSGS, suggesting impaired fatty acid oxidation and possible mitochondrial dysfunction. Mutations in *SGPL1*, the gene which encodes S1P lyase, have been reported to be associated with steroid-resistant nephrotic syndrome [8,9,83], which manifests histologically with FSGS and diffuse mesangial sclerosis [84]. *Sgpl* knockout mice were shown to recapitulate many features of human renal disease [9]. Using microarray analysis on glomeruli isolated from patients with FSGS of the NEPTUNE cohort, we previously demonstrated altered expression of genes involved in cholesterol and free fatty acid homeostasis [4], supporting the hypothesis that dysregulation of cholesterol homeostasis contributes to the pathogenesis of FSGS. Additionally, we previously reported an important role of sphingomyelin phosphodiesterase acid-like 3b (SMPDL3b) in the recurrence of FSGS after transplantation [85]. We demonstrated that the expression of SMPDL3b is significantly decreased in podocytes from patients with recurrent FSGS. The role of SMPDL3b in glomerular diseases will be discussed in detail in the section below.

Therefore, similarly to DKD, modulating the sphingolipid content in glomeruli may also represent a valid strategy to prevent or treat podocyte injury in FSGS.

### Role of SMPDL3b in the kidney

SMPDL3b is a glycosylphosphatidylinositol (GPI)anchored protein [86,87] with reported roles in inflammatory processes [88] and in kidney diseases [6,40,85]. Recently, the crystal structure of murine SMPDL3b was revealed and helped to identify possible substrates for SMPDL3b, which included CDP-choline, ATP and ADP [89]. Sphingomyelin could also be a potential substrate for SMPDL3b.

In macrophages, deficiency of *Smpdl3b* has been shown to cause alterations in the membrane lipid composition and changes in its fluidity [88,90]. The same study demonstrated that *Smpdl3b* knockdown results in enhanced responsiveness to Toll-like receptor stimulation (TLR4) and increased inflammatory response. SMPDL3b expression has also been found in pancreatic zymogen granules [91], in plasma protein-depleted cerebrospinal fluid [92], in saliva exosomes [93], in human milk [94], liver [95], and hepatocellular carcinoma [96], suggesting a diverse function of SMPDL3b in different tissues and organs.

In the kidney, we previously reported that SMPDL3b is a resident in lipid raft domains [85]. In the same study, we demonstrated that kidney biopsies of patients with recurrence of FSGS express less SMPDL3b, while overexpression of SMPDL3b in podocytes can prevent actin cytoskeleton disruption and apoptosis. Furthermore, we reported that rituximab, an anti-CD20 monoclonal antibody targeting B cells, binds to SMPDL3b, thereby protecting podocytes from cytoskeleton disruption and apoptosis induced by treatment with sera from patients with recurrent FSGS [85]. Similarly, SMPDL3b protein loss has been reported in xenotransplants, where rituximab pretreatment maintained levels of SMPDL3b [97]. Additionally, adriamycin-induced nephropathy in rats was associated with reduced expression of SMPDL3b, which was also prevented by rituximab [98].

In contrast, SMPDL3b expression is elevated in glomeruli from patients with DKD, as well as in animal models of DKD, where SMPDL3b expression in kidney cortices of 24-week-old diabetic *db/db* mice was found to be almost three-fold higher when compared to controls using a transcriptomic approach [39]. We previously demonstrated that, in podocytes, SMPDL3b binds to the soluble urokinase plasminogen activator receptor (suPAR) (40), which is reported to be elevated in sera from patients with FSGS and DKD among other causes of CKD [40,99,100]. Furthermore, FSGS sera-treated podocytes showed decreased expression of SMPDL3b in association with increased cortical actin and loss of stress fibers, while podocytes treated with DKD serum demonstrated increased expression of SMPDL3b in association with actin reorganization in cell blebs [40].

Moreover, an excess of SMPDL3b in human podocytes may cause the displacement of insulin receptor isoforms from caveolin-1-rich domains of the plasma membrane in a C1P-dependent manner, resulting in impaired ability to phosphorylate AKT thus promoting podocyte injury *in vitro* and development of DKD *in vivo* [6]. In addition, we demonstrated that overexpression of SMPDL3b in human podocytes causes suppression of protein kinase B and activation of p70S6 kinase phosphorylation, through its binding to both insulin receptor isoforms, IR-A and IR-B and caveolin-1, suggesting a novel role for SMPDL3b as a modulator of insulin signaling in podocytes [6]. Indeed, we demonstrated that SMPDL3b can interfere with the binding of insulin receptor B to caveolin-1 while facilitating insulin receptor A binding to caveolin-1, which may be responsible for increased insulin-stimulated p70S6 kinase phosphorylation. Thereby, overexpression of SMPDL3b is associated with reduced levels of C1P in human podocytes *in vitro* and in kidney cortices *in vivo* [6], while podocyte-specific deficiency of SMPDL3b in diabetic *db/db* mice results in reduced proteinuria and improved renal outcome. The idea that SMPDL3b may regulate the availability of bioactive sphingolipids such as C1P in podocytes is indeed intriguing. However, if SMPDL3b may have a direct phosphatase activity and convert C1P to ceramide remains to be investigated. Our further studies demonstrated that SMPDL3b interacts with ceramide kinase (CERK) and binds C1P *in vitro* and that SMPDL3b expression positively correlates with CERK expression [7].

Interestingly, single dose irradiation of human podocytes results in a time-dependent decrease of SMPDL3b protein expression in association with cortical actin remodeling, while overexpression of SMPDL3b in human podocytes yields a protective effect from radiation injury [101]. Pretreatment with rituximab mitigated radiation-induced cytoskeletal changes and increased expression levels of SMPDL3b [101]. In contrast, a single dose of radiation applied to human immortalized glomerular endothelial cells results in increased levels of SMPDL3b and decreased levels of C1P [102], while treatment with exogenous C1P or genetic knocking down of SMPDL3b partially protects glomerular endothelial cells. Notably, inhibition of NOX1 seems sufficient to restore normal expression of SMPDL3b and to reduce radiation-induced damage of glomerular endothelial cells. For a more detailed review on the role of sphingolipids in renal oncology, the reader is referred to [103].

Although these reports provide clues to the functions of SMPDL3b in various cells and organs, a better understanding of the role of SMPDL3b in health and disease is still needed. Nevertheless, SMPDL3b seems to be an attractive therapeutic target, at least for the treatment of glomerular diseases.

## Targeting sphingolipids in DKD and FSGS

A role for sphingolipids as modulators of podocyte function in DKD, FSGS and other glomerular diseases is an emerging concept. Increasing evidence suggests an involvement of sphingolipid metabolism in the development and progression of glomerular diseases. It has become clear that targeting sphingolipids may be beneficial for the treatment of DKD and FSGS.

One option to target sphingolipids is through the manipulation of S1P receptors (S1PRs). Treatment with FTY720, or fingolimod, a non-selective S1PR agonist, or SEW2871, a selective agonist of S1PR1, was shown to reduce urinary albumin excretion as well as urinary levels of the pro-inflammatory cytokine tumor necrosis factoralpha in mouse and rat models of STZ-induced DKD [104], suggesting that targeting kidney S1PR1 may represent a therapeutic intervention for the treatment of DKD. Additionally, treatment with berberine improved renal injury in STZ-induced diabetic rats via downregulation of S1PR2 [105] and the use of the S1PR2 specific antagonist LTE-013 showed improved insulin resistance in human and rat hepatocytes [106], suggesting the possibility that S1PR2 inhibition may prove useful for the treatment of diabetes and its related complications. Another study demonstrated that inhibition of ceramide accumulation with myriocin in Otsuka Long Evans Tokushima fatty rats and HFD-fed mice ameliorates albuminuria and histologic features of DKD [107]. Finally, our studies demonstrated that exogenous C1P treatment or podocyte-specific inhibition of SMDP3Lb results in reduced proteinuria, improves renal outcome and restores insulin receptor signaling in diabetic *db/db* mice [6].

While the therapeutic use of active sphingolipids or modulators of S1P receptor activation remains of high interest and deserves further study, the possibility to target sphingolipid related enzymes remains an attractive opportunity. Among them, SMPDL3b may surely represent an attractive target. As no drug is yet available to directly agonize and antagonize the function of SMPDL3b, repurposing strategies with rituximab or other anti-CD20 antibodies that recognize the same epitope could and should be considered. Our retrospective study clearly suggested that a single dose of rituximab administered in the pre-transplant setting may be sufficient to prevent the recurrence of proteinuria after transplantation in patients with FSGS [85]. Observational studies have demonstrated that rituximab is a safe and effective treatment option in patients with steroid-dependent or frequently relapsing nephrotic syndrome, including minimal change disease and FSGS. While randomized controlled trials are needed, rituximab has been shown to prevent recurrences and reduce the need for immunosuppressive therapy [108,109]. A recent meta-analysis on the use of rituximab in clinical settings suggests an additional benefit of rituximab if added to the standard therapy in adults with FSGS [110]. Therefore, rituximab may represent a potentially effective agent for the treatment of some glomerular diseases. Whether these beneficial effects are due to B lymphocyte depletion or to stabilization of SMPDL3b expression and function cannot be discerned in treated patients and requires further experimental studies with humanized mouse models.

Another reported therapy targeting sphingolipids is enzyme replacement therapy (ERT), which currently represents a standard of therapy for Fabry disease, or lipid storage disease. In a recent case report of a Japanese man with FSGS and low activity of alpha-galactosidase (with mutation in M296I), ERT in association with immunotherapy with steroids and cyclosporine A [111] improved proteinuria levels, while in another case report of an obese male with histologically proven FSGS and low activity of alpha-galactosidase (with missense mutation in R310Q) no improvement in renal function despite ERT was described [112]. Thus, while ERT may be a very promising therapy for the treatment of lipid storage disease, its therapeutic potential in the treatment of patients with FSGS warrants further investigation.

## Concluding Remarks

**Figure 2:**
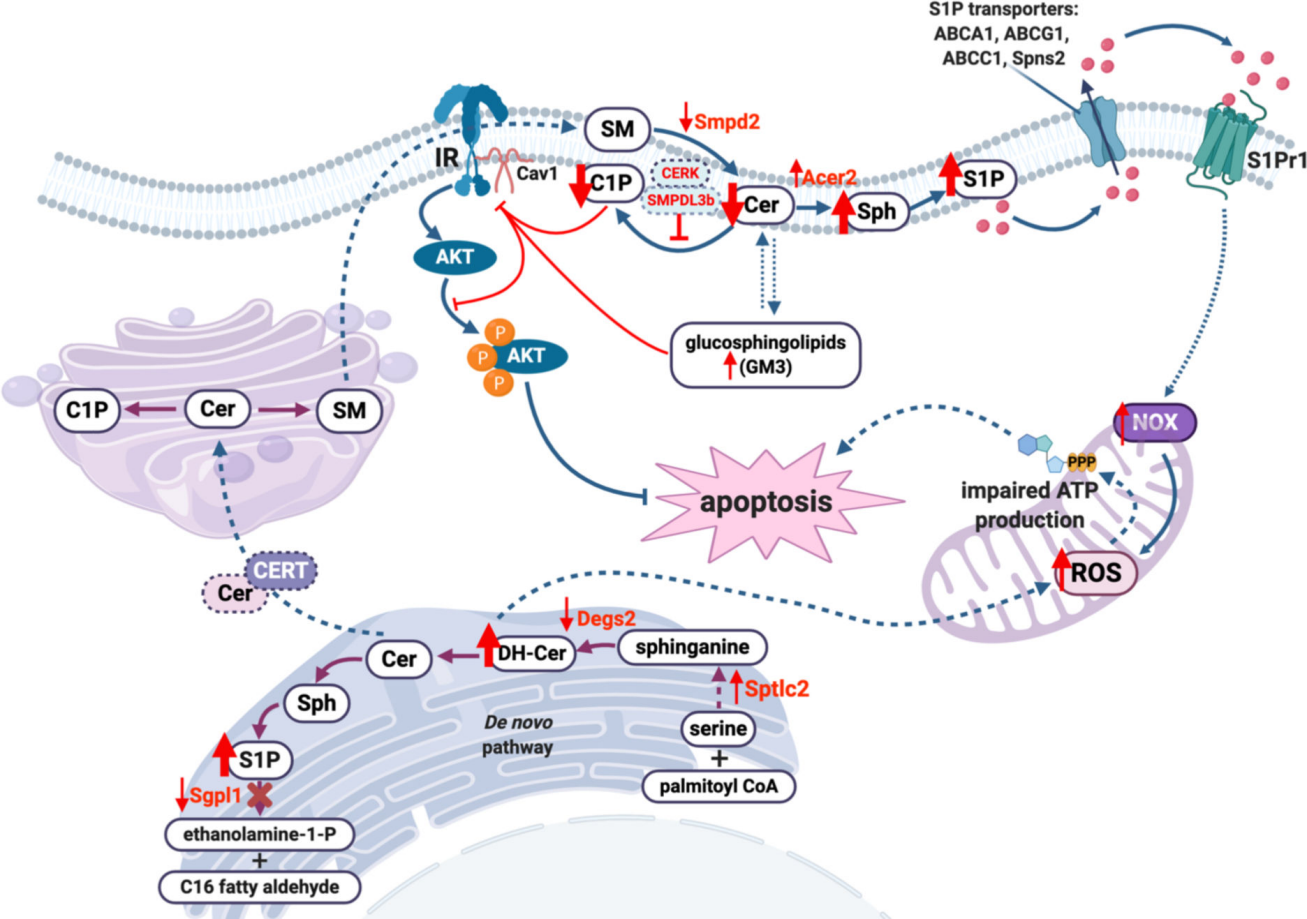
Suggested mechanism of the dysregulation of the sphingolipid machinery in glomerular diseases. The data summarized are related to the events in kidney cortex only. Decreased activity of desaturase (Degs2) results in the accumulation of dihydroceramides (DH-Cer) and causes accumulation of reactive oxygen species (ROS), which impairs ATP production and leads to apoptosis. Decreased activity of sphingosine-1-phosphate lyase 1 (Sgpl1) results in the accumulation of sphingosine-1-phosphate (S1P). At the plasma membrane, decreased activity of sphingomyelin phosphodiesterase 2 (Smpd2) affects ceramide (Cer) production, while elevated activity of alkaline ceramidase 2 Acer2) increases levels of sphingosine (Sph) and, as a consequence, S1P. Overproduction of S1P results in increased S1P efflux via S1P transporters (such as ATP-binding cassette transporters ABCA1, ABCG1, ABCC1 and S1P transporter Spns2), where S1P acts as a paracrine factor and activates S1P receptors (primarily, S1P receptor 1, S1Pr1), leading to overproduction of NAPDH oxidase (NOX), increased ROS production and apoptosis. Overexpression of sphingomyelin phosphodiesterase acid-like 3b (SMPDL3b) blocks ceramide kinase (CERK) activity and the conversion of ceramide to ceramide-1-phosphate (C1P), which affects interaction between insulin receptor (IR) and caveolin-1 (Cav1), leading to decreased protein kinase B (AKT) phosphorylation and apoptosis. Accumulation of the ganglioside GM3 at the plasma membrane has a similar effect, causing displacement of IR from Cav1 and impaired AKT phosphorylation.

In this review, we highlighted new research trends and scientific knowledge acquired within the past few years indicating that sphingolipids are key players contributing to the pathogenesis and progression of glomerular diseases such as DKD and FSGS (Figure 2). Studies suggest that SMPDL3b, S1P lyase, C1P, S1P and S1P receptors are valid and important targets for the development of novel therapeutic therapies for glomerular diseases. Manipulation of the S1P signaling pathway, particularly modulation of S1P receptors and/or sphingosine kinases is evolving as an attractive therapeutic strategy to slow the progression of kidney diseases. An important question, which needs to be further addressed in detail, is the contribution of altered sphingolipid metabolism to the pathogenesis of glomerular diseases. Indeed, while many reports describe changes in sphingolipid levels and species at disease onset, it remains unclear if these changes are in fact pathogenic. Moreover, because of their biophysical properties, sphingolipids are less capable of moving freely from one compartment to another inside of a cell. Thus, a better understanding of the relationship between the localization and function of sphingolipids in different cell compartments is needed as it may explain some of the conflicting reports in the literature and further our understanding of the role of sphingolipids in the pathogenesis and progression of glomerular diseases.

## Highlights

- Sphingolipids and related enzymes modulate podocyte function in both DKD and FSGS.
- Ceramide, sphingosine, ceramide-1-phosphate and sphingosine-1-phosphate may directly contribute to the pathogenesis of glomerular diseases.
- Sphingomyelin phosphodiesterase acid-like 3b (SMPDL3b) is a novel enzyme involved in the development of podocyte injury in DKD and FSGS.
- Therapeutic strategies that target cellular sphingolipids might be protective in DKD, FSGS, and more broadly chronic kidney diseases.
